# Review of Neurostimulation Therapies for Obstructive Sleep Apnea: Hypoglossal Nerve Stimulation and Beyond

**DOI:** 10.3390/jcm14155494

**Published:** 2025-08-04

**Authors:** Patrícia dos Santos Cé, Maria Eduarda Schiestl Melo, Alan Alves Machado, Sarah Eden Ridge, Thomaz Fleury Curado

**Affiliations:** 1 Department of Dentistry, University Hospital Polydoro Ernani de São Thiago, Federal University of Santa Catarina, Florianópolis 88036-800, Brazil; 2 Department of Dentistry, Federal University of Santa Catarina, Florianópolis 88036-800, Brazil; mariaeduardaschiestl@gmail.com (M.E.S.M.); alanmachado96@gmail.com (A.A.M.); 3 Department of Otorhinolaryngology—Head and Neck Surgery, Case Western Reserve School of Medicine, University Hospitals Cleveland Medical Center, Cleveland, OH 44106, USA; sarah.ridge@uhhospitals.org (S.E.R.); thomaz.fleurycurado@uhhospitals.org (T.F.C.)

**Keywords:** obstructive sleep apnea, hypoglossal nerve stimulation, airway management

## Abstract

Obstructive sleep apnea (OSA) is a sleep-related respiratory disorder characterized by partial or complete obstruction of the upper airway, typically resulting in a decrease in arterial oxygen saturation and repeated awakenings from sleep. It is the most common sleep-related respiratory disorder, affecting 9% to 38% of adults. OSA is associated with loss of tone, improper contraction of the tongue, and pharyngeal dilator muscles of the upper airway during sleep. The gold-standard treatment for moderate-to-severe OSA is continuous positive airway pressure (CPAP). However, many patients have poor long-term compliance with CPAP. Stimulation of the upper airway with electrical activation of the hypoglossal nerve has emerged as a promising treatment for patients with moderate-to-severe OSA who have failed CPAP therapy. Objectives: The present paper aims to review the literature regarding neurostimulation for the treatment of OSA. Conclusions: Hypoglossal nerve stimulation (HNS) has shown favorable success and low morbidity in the management of moderate-to-severe OSA.

## Introduction

Obstructive sleep apnea (OSA) is a sleep disorder that is characterized by repetitive, partial, or complete obstruction of the upper airway, typically leading to reduced blood oxygen saturation and sleep awakenings [1,2]. It is the most common type of sleep-related respiratory disorder, affecting between 9% and 38% of the adult population [3]. OSA is associated with loss of tone and improper contraction of the tongue and upper airway muscles during sleep [2]. The standard treatment for moderate-to-severe OSA is continuous positive airway pressure (CPAP) [1], although adherence to and long-term use of CPAP are often low. Alternative treatment options include customized intraoral appliances (IOAs), upper airway surgery (UAS), and maxillomandibular advancement (MMA) surgery. Recently, upper airway stimulation with electrical activation of the hypoglossal nerve has emerged as a promising option for patients with moderate-to-severe OSA who have not had success with CPAP therapy. This approach has shown favorable outcomes with low morbidity [1].

**Figure 1:**
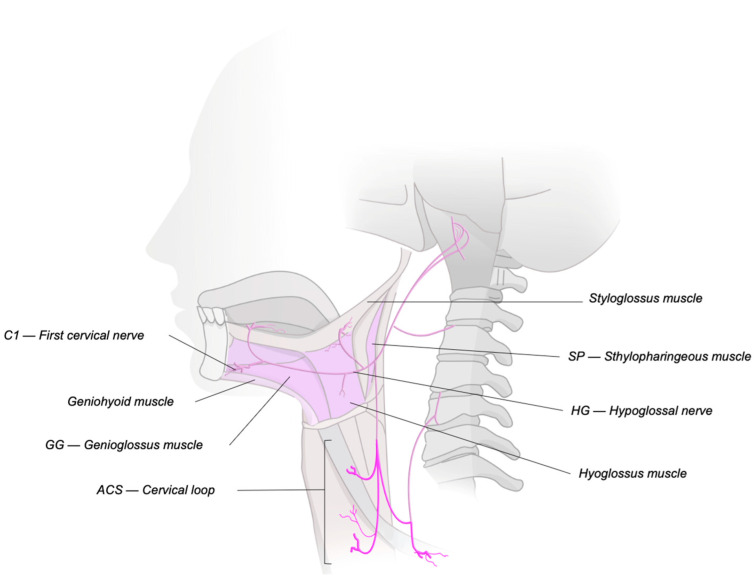
Innervation of the upper airway.

To better understand the mechanism of OSA, it is crucial to first grasp the anatomy of the upper airway (Figure 1). Regarding the shape of the pharynx, in 1996, Leiter proposed that the tongue plays a key role in maintaining pharyngeal patency. He described an elliptical pharyngeal lumen, where the major axis is oriented laterally and the minor axis is oriented anteroposteriorly [4]. He describes that tongue protrusion leads to a more significant improvement in patency for patients with elliptical pharyngeal collapse, as opposed to concentric collapse. This idea is supported by physiological studies that show much greater reductions in pharyngeal collapsibility in patients with predominantly anteroposterior pharyngeal collapse, rather than lateral collapse, during sleep endoscopy. However, these responses might be less pronounced in individuals with a larger soft palate, suggesting that both the tongue and bony structures influence these outcomes. According to Fleury et al., the shape of the pharyngeal lumen in both the retropalatal and retroglossal segments, along with the tongue’s configuration under stimulation, can help predict the response to this treatment [5]. Many apneic patients show retropalatal collapse as the main site of airway obstruction during sleep. Patients who respond to hypoglossal nerve stimulation tend to show greater retropalatal expansion than non-responders, even when retroglossal expansion is similar in both groups. The response to hypoglossal nerve stimulation may depend on the interaction between the palatoglossus muscle and the available space within the maxillomandibular complex [5].

Studies indicate that obstructive respiratory events occurring during sleep may be linked to reduced activity of the pharyngeal dilator muscles, including the genioglossus muscle, which is innervated by the hypoglossal nerve (CN XII) [6,7]. As a result, electrical stimulation of this nerve has emerged as a promising solution to prevent upper airway collapse [8]. When stimulated, the tongue triggers the activation of other pharyngeal muscles and increases the volume of the upper airway. Selective stimulation of the trunk of the hypoglossal nerve, which has a multifascicular structure, can activate the genioglossus, along with other pharyngeal dilator muscles, working to prevent pharyngeal collapse in individuals with OSA [9].

The introduction of hypoglossal nerve stimulation (HNS) has revolutionized surgical management for OSA and remains the only surgical intervention that works by restoring neuromuscular activity, rather than altering upper airway anatomy. It is a highly effective treatment, with improved patient adherence when compared to CPAP [10]. Research suggests that HNS has a similar efficacy in improving sleep efficiency, quality of life measures, and reducing the apnea–hypopnea index (AHI) when compared to CPAP [10].

**Figure 2:**
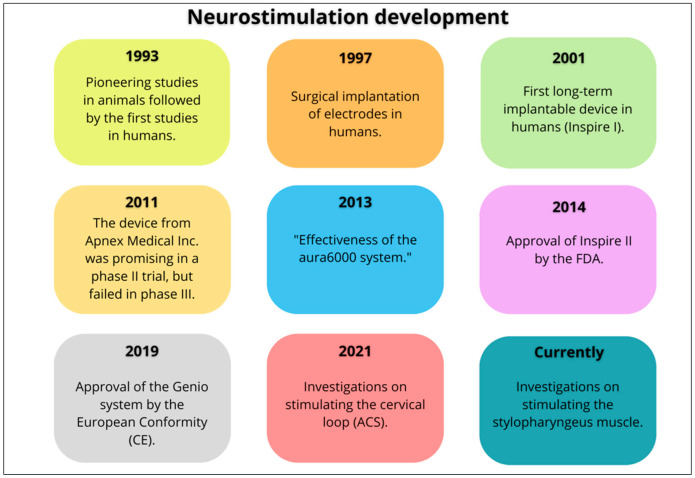
A timeline of developments in upper airway neurostimulation.

HNS has been investigated as a treatment for OSA since the late 1980s, initially using surface electrodes [11] and later using implanted electrodes [12] (Figure 2). HNS was first approved by the FDA in 2014, based on the study entitled “Stimulation Treatment for Apnea Reduction (STAR) trial,” which used the Inspire^®^ device. This study, published by Strollo et al. [13], demonstrated significant improvements, both objective and subjective, in patients with moderate-to-severe OSA. The study involved 126 participants with an AHI ranging from 15 to 65 events per hour, who were intolerant to positive airway pressure therapy. Of the 126 initial participants, 97 (78%) returned for the 5-year follow-up. Both AHI and oxygen desaturation index (ODI) showed significant reductions from baseline after 12 months, and these improvements remained stable at 36 and 60 months. After 5 years, 75% of participants maintained a reduction in their AHI of more than 50%, with 44% having an AHI of less than 5, and 78% having an AHI of less than 15. The analysis revealed an average AHI of 15.1 at the 5-year mark, with a response rate of 63%, which is similar to the rate observed at 12 months. Multivariate analysis indicated that a lower baseline ODI was predictive of a better AHI response after 5 years. Additionally, improvements in the Functional Outcomes of Sleep Questionnaire (FOSQ) and the Epworth Sleepiness Scale (ESS) persisted, with 67% reporting normal FOSQ scores and 78% showing normal ESS scores after 5 years. Partner reports revealed a significant reduction in snoring, and 80% of participants continued to use the device regularly. While there were no major changes in sleep stage distribution, the number of awakenings decreased. Regarding adverse events, 6% of participants experienced complications, most of which were resolved with adjustments over time [14].

In 2019, another HNS device was approved in the European Community—the Genio^®^ system—which was designed for bilateral stimulation, based on the Nyxoah BLAST OSA study (Bilateral Hypoglossal Nerve Stimulation for the Treatment of Obstructive Sleep Apnea). This prospective study evaluated the safety and performance of the Genio^®^ system across seven centers in France and Australia. It concluded that the Genio™ system effectively reduced the severity of obstructive sleep apnea (OSA) and enhanced quality of life, with no complications related to the device. The results were comparable to those of previously published HNS studies, but the Genio system involved fewer implanted components and featured a simpler stimulation algorithm [15]. Currently, besides the Inspire (Inspire Medical Systems Inc., Maple Grove, MN, USA) and Nyxoah (Genio, Moint San-Guibert, Belgium)) systems, there is also the Aura6000 (Livanova, UK). The differences between each system are detailed in Table A1, with all devices having similar clinical outcomes [15,16,17].

**Figure 3:**
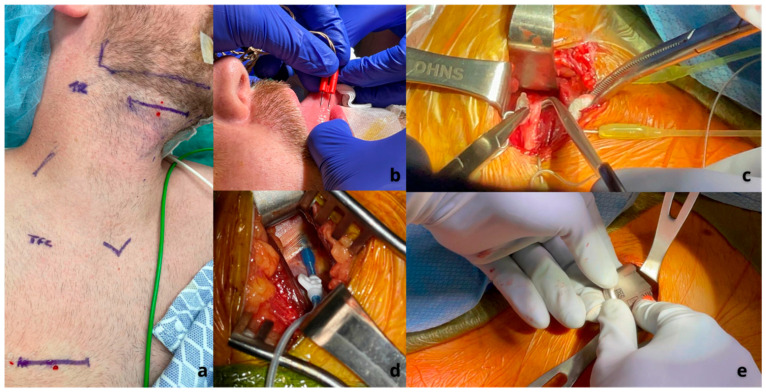
Sequence of HNS implantation using the Inspire^®^ system. Source: Archive of Thomaz Fleury, MD. (**a**) Identification of landmarks and incision planning. (**b**) Insertion of monitoring electrodes into the hyoglossus muscle. (**c**) Placing the cuff of the stimulation lead around the hypoglossal nerve. (**d**) Insertion of the sensing lead between the internal intercostal and external intercostal muscles. (**e**) Placing the implantable pulse generator into the subcutaneous pocket in the chest.

The Inspire^®^ upper airway stimulation system (Inspire Medical Systems Inc.) consists of a breathing sensor, a programmable pulse generator, and stimulating electrodes. The sensor is placed between the internal and external intercostal muscles and detects the movements of the chest during breathing. These data are then analyzed by the pulse generator. The generator is typically implanted in the upper right side of the chest, just under the fascia of the pectoralis major muscle, and it delivers stimulation that is synchronized with the breathing cycle to the stimulation electrode. This electrode is placed on the anterior branches of the hypoglossal nerve (which controls the tongue) and the first cervical spinal nerve (C1). When these nerves are stimulated, they cause the tongue to move forward by activating the genioglossus muscle. Additionally, stimulating the C1 nerve results in the hyoid bone moving upward and forward, which further aids in opening the airway. Studies have shown that the benefits of this stimulation go beyond the tongue base and improve airway patency up to the level of the palate [1] (Figure 3).

The ImThera Aura6000 (Livanova) is a selective hypoglossal nerve stimulation system consisting of a device that includes a generator, an implant with six contact electrodes, and a connecting wire. Unlike other systems, this device does not use an intercostal respiratory sensor, which helps reduce the risk of pulmonary complications. The generator is typically implanted beneath the right clavicle in a subcutaneous pocket and connects to an electrode wrapped around the hypoglossal nerve via a subcutaneous pathway. Functionality is tested through telemetry, with confirmation through the movement of the tongue. The device’s battery is rechargeable via transcutaneous charging with an external charger. The generator has memory to record charging and usage data, which can be accessed remotely to adjust the stimulation parameters to prevent airway obstruction during sleep [18].

Unlike the Inspire and Aura6000, the Nyxoah—Genio stimulator works through bilaterally stimulating the hypoglossal nerve. It consists of two sets of paired stimulating electrodes and a central receiver antenna. The electrodes are placed on flexible legs, which adapt to the anatomical variations in the hypoglossal nerve branches and allow for the movement of the genioglossus muscle. The design of the stimulator enables bilateral placement of the electrodes over the terminal branches of the hypoglossal nerve, stimulating both genioglossus muscles. This is the only implantable part of the Genio system, which is activated by wireless power transmitted from an activation chip that the patient wears while sleeping. Each morning, the patient removes the chip from an adhesive patch, disposes of the patch, and recharges the chip. The chip’s memory stores the specific stimulation parameters set by their physician [19].

A key requirement for considering HNS is the completion of drug-induced sleep endoscopy (DISE), which is a diagnostic procedure that helps identify the exact site of airway collapse (such as retropalatal or retrolingual), the collapse pattern (concentric, anteroposterior, or lateral), and the severity (complete or partial) of the obstruction. Sleep endoscopy is particularly valuable in ruling out complete concentric collapse (CCC) at the level of the soft palate, which is considered the most significant contraindication for HNS. This pattern of obstruction is relatively common, affecting about 20% to 25% of patients with OSA who are intolerant to CPAP. In these cases, even though other criteria might allow for device implantation, the anatomical collapse pattern suggests a high risk of treatment failure; therefore, HNS therapy should not be recommended in these circumstances [20].

**Figure 4:**
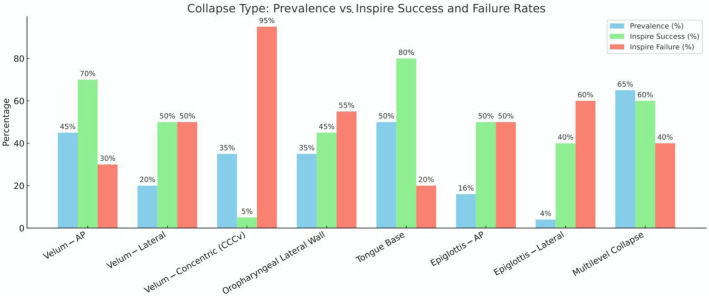
Prevalence of upper airway collapse patterns on drug-induced sleep endoscopy (DISE) and estimated rates of Inspire hypoglossal nerve stimulation success and failure for each pattern.

Figure 4 illustrates the estimated prevalence of eight common airway collapse types observed on DISE, alongside the expected success and failure rates of Inspire therapy for each. As anticipated, velum anteroposterior (AP) and tongue base collapse are among the most frequently observed patterns (prevalence ~45–50%) and are associated with the highest HNS success rates (~70–80%). In contrast, CCC remains both common (~35%) and notably refractory to Inspire therapy, with success rates below 10%, confirming its role as a primary contraindication. Collapse involving the oropharyngeal lateral walls and epiglottis showed more variable responses to Inspire. Despite their significant prevalence (~30–35%), these patterns demonstrated lower and more unpredictable success rates (~40–50%), likely due to their dynamic, multidirectional nature and their partial independence from genioglossus-mediated airway patency. Multilevel collapse, although highly prevalent (~65%), showed intermediate success rates (~60%) [13,21,22,23].

These findings underscore the importance of proper phenotyping and patient selection and highlight the necessity of incorporating DISE into preoperative evaluation to optimize therapeutic outcomes. They also emphasize the opportunity for advancing beyond traditional single-muscle stimulation paradigms. As shown, collapse types with suboptimal outcomes are common and may benefit from next-generation approaches involving tailored multi-muscle or multi-site neurostimulation strategies, including bilateral or novel targets such as the ansa cervicalis and glossopharyngeal nerve.

In 2021, Kent et al. investigated whether ansa cervicalis stimulation (ACS) as a means to contract the sternothyroid muscle could increase pharyngeal patency in those with OSA [24]. This study investigated the effects of ACS and HNS, both individually and combined, on pharyngeal patency during sedation with propofol. The findings revealed significant increases in maximum inspiratory airflow and highlighted the potential of ACS as a new strategy for respiratory neurostimulation (RNS) in treating OSA. The study demonstrated that ACS targeting the sternothyroid muscle independently increased inspiratory airflow in OSA patients during DISE, offering a potential rescue therapy when unilateral hypoglossal nerve stimulation does not achieve the expected results. To enhance HNS, future RNS strategies must ultimately expand beyond the hypoglossal nerve. The cervical loop branch to the sternothyroid muscle is easily accessible, and the strong stimulation responses observed in this study suggest that ACS significantly improves pharyngeal patency, working synergistically with HNS. ACS leverages the well-established benefits of caudal traction on pharyngeal permeability and represents a notable departure from current surgical treatments for OSA, which primarily focus on ventrally repositioning the tongue and other soft tissues of the pharynx. However, further studies are essential to fully understand the role of ACS within the broader landscape of surgical and neurostimulation treatment strategies [24].

Finally, glossopharyngeal nerve stimulation has also been studied in the treatment of OSA. The stylopharyngeus muscle inserts medially into the lateral wall of the oropharynx, positioned between the superior and middle constrictor muscles. It is innervated by the glossopharyngeal nerve, which also contributes to the motor branches of the pharyngeal plexus that innervate the constrictor muscles. To investigate the hypothesis proposed by Guilleminault et al., which suggests that the coactivation of the stylopharyngeus and pharyngeal constrictor muscles stiffens and laterally pulls the oropharyngeal wall to stabilize it during inspiration, Kent et al. (2025) introduced a new technique for percutaneous glossopharyngeal nerve stimulation (GNS) [25,26]. The results from Kent et al. [26] supported Guilleminault’s hypothesis that the lateral movement of the oropharyngeal wall improves upper airway patency, suggesting that GNS could be an effective respiratory neurostimulation strategy for OSA. It was observed that GNS resulted in the lateral movement of the oropharyngeal wall, indicating a strong contraction of the stylopharyngeus muscle, possibly along with additional activation of the pharyngeal constrictor muscles. While direct confirmation of stylopharyngeus contraction was not possible, no other muscles in the parapharyngeal space are expected to pull the oropharyngeal wall laterally in this manner [26].

In a 2024 systematic review by Alrubasy et al. [27], three HNS devices—Apnex, Inspire, and ImThera—were compared. The review evaluated each device’s efficacy individually, examining outcomes over both short-term (≤1 year) and long-term (>1 year) periods. The study included a total of 30 papers, with 26 of these being single-arm studies involving 549 middle-aged overweight patients. Four randomized controlled trials (RCTs) included 273 participants. The results indicated that HNS is both an effective and safe treatment option. The Inspire device demonstrated significant improvements, reducing the AHI by 20.14 events per hour in the short term and 15.91 events per hour in the long term. Additionally, the ODI decreased by 14.16 events per hour in the short term and 12.95 events per hour in the long term. Patient-reported outcomes showed a reduction in ESS scores by 5.02 in the short term and 4.90 in the long term, while the FOSQ scores improved by 3.58 in the short term and 3.28 in the long term. Both the Apnex and ImThera devices showed similar improvements, though to a lesser extent. The authors concluded that hypoglossal nerve stimulation is a safe and effective treatment for patients with OSA, demonstrating high adherence and satisfaction rates. However, they emphasized the need to refine patient selection criteria to include a broader range of individuals with OSA [27].

Wesson et al. [28] aimed to systematically outline how polysomnography (PSG) and AHI are reported in prospective studies that involve unilateral hypoglossal nerve stimulation. Fifteen studies met the inclusion criteria, consisting of fourteen prospective cohort studies and one randomized controlled trial. Titration PSG was the primary method used to gather data in five of the studies, while only three studies relied solely on non-titration PSG to report outcomes. Three studies combined data from two or more sleep studies to present a single AHI. Among the fifteen studies, the non-titration AHI was the most frequently reported (five studies), while titration AHI was used in just one study. Additionally, five studies did not specify the type of AHI used to assess treatment effectiveness. The sleep studies and corresponding AHI values reported in the studies varied significantly. Due to this high level of variability, future research would greatly benefit from the consistent use of a standardized AHI to report outcomes related to HNS [28].

Pordzik et al. [29] aimed to directly compare first-line positive airway pressure (PAP) therapy for OSA, particularly auto-adjusting PAP (aPAP), with second-line HNS therapy by using standard PSG-related parameters and patient-reported outcomes in comparable groups. The study included 20 patients in the HNS group (mean age 57.30 ± 8.56 years; 6 females) and 35 patients in the aPAP group (mean age 56.83 ± 9.20 years; 9 females). All participants met the current guideline criteria for HNS treatment. The groups were compared using analysis of covariance with inverse propensity score weighting. Propensity scores did not differ between the groups. The pre-treatment AHI and oxygen desaturation index (ODI) were similar between both groups. After 413.6 ± 116.66 days of treatment in the HNS group and 162.09 ± 140.58 days in the aPAP group, the AHI was significantly higher in the HNS group compared to the aPAP group. However, the ESS score was significantly lower post-treatment in the HNS group compared to the aPAP group. These findings provide novel real-world data, and further research is needed to refine the parameters for titrating HNS neurostimulation and better understand the factors influencing HNS adherence [29].

It is also important to mention that sponsor-related bias may be present in publications related to HNS [30,31]. Looking forward, there is a need for further studies from authors without connections to companies producing stimulators.

Neurostimulation, particularly through hypoglossal nerve stimulation, has undoubtedly emerged as an effective form of airway management for the treatment of obstructive sleep apnea. By targeting the hypoglossal nerve, these devices aim to restore the necessary neuromuscular activity to prevent airway collapse during sleep, offering a viable alternative to traditional surgical treatments that focus on anatomical correction. The growing field of neurostimulation expands beyond the hypoglossal nerve, with innovative approaches like ansa cervicalis stimulation and glossopharyngeal nerve stimulation showing potential to enhance pharyngeal patency and airflow, working synergistically with hypoglossal nerve stimulation. These advancements suggest that neurostimulation not only helps manage airway obstruction but can also complement existing therapies, improving overall treatment outcomes. Nevertheless, more research is needed to refine these techniques and better understand how they can be integrated into comprehensive treatment plans for airway management in obstructive sleep apnea.

## Abbreviations

The following abbreviations are used in this manuscript:

OSA	Obstructive Sleep Apnea
CPAP	Continuous Positive Airway Pressure
IOAs	Intraoral Appliances
UAS	Upper Airway Surgery
MMA	Maxillomandibular Advancement
HNS	Hypoglossal Nerve Stimulation
AHI	Apnea–Hypopnea Index
ODI	Oxygen Desaturation Index
STAR	Stimulation Treatment for Apnea Reduction
FOSQ	Functional Outcomes of Sleep Questionnaire
ESS	Epworth Sleepiness Scale
C1	First Cervical Spinal Nerve
DISE	Drug-Induced Sleep Endoscopy
CCC	Complete Concentric Collapse
ACS	Ansa Cervicalis Stimulation
RNS	Respiratory Neurostimulation
GNS	Glossopharyngeal Nerve Stimulation
PSG	Polysomnography
PAP	Positive Airway Pressure

## Appendix A

**Table A1:** Comparison between the Inspire^®^, Nyxoa Genio^RM^, and LivaNova aura 6000^RM^ systems.

	Selective Stimulation		Targeted Stimulation
Laterality	Unilateral	Bilateral	Unilateral
Device	Inspire ^®^	Nyxoah Genio System ^TM^	LivaNova aura6000 ^TM^
Age	>18 years >13 years with Down Syndrome	>18 years	>22 years
AHI	15–100 events/hour (adult) 15–50 events/hour (pediatric)	15–65 events/hour	20–65 events/hour
BMI	<40 kg/m^2^	<35 kg/m^2^	<35 kg/m^2^
Central and Mixed Apneas	Central and/or mixed apnea index < 25 % of AHI	Central and/or mixed apnea index < 25 % of AHI	Central and/or mixed apnea index < 25 % of AHI
DISE	Required to exclude CCC	Not necessary; indicated for patients with CCC	Not necessary
Implantable Device	3 pieces (IPG, stimulation electrode, sensor electrode)	1 piece (stimulation electrode)	2 pieces (IPG, stimulation electrode)
Implant Incisions	2	1	2
Stimulation Electrode	Medial and distal branches of the HN	Medial and distal branches of the HN	Main Branch of HN
Respiratory Cycle Dependence	Yes (intercostal sensor, pressure sensor)	Stimulation cycle dependent	Not dependent
Battery Life Expectancy	~10.7 years	External Battery and IPG	~10–15 years
Charging	Not required	Daily, external battery	2 days for 10–15 min
Battery Replacement	IPG	Not required	IPG
Programming	Telemetry	External programming	Telemetry
MRI Compatibility	1.5 T (FDA)	3.0 T	Not compatible

## References

[1] Veugen C.C.A.F.M., Dieleman E., Hardeman J.A., Stokroos R.J., Copper M.P. (2023). Upper Airway Stimulation in Patients with Obstructive Sleep Apnea: Long-Term Surgical Success, Respiratory Outcomes, and Patient Experience. Int. Arch. Otorhinolaryngol..

[2] Tong J.Y., Gocal W.A., Haft S.J. (2024). Adverse Events Associated with Device Assisted Hyoid and Tongue Base Suspension for Obstructive Sleep Apnea. Am. J. Otolaryngol..

[3] Chang C.P., Poomkonsarn S., Giannakopoulos H., Ma Y., Riley R., Liu S.Y. (2023). Comparative Efficacy of Obstructive Sleep Apnea Patients Undergoing Multilevel Surgery Followed by Upper Airway Stimulation versus Isolated Upper Airway Stimulation. J. Oral Maxillofac. Surg. Off. J. Am. Assoc. Oral Maxillofac. Surg..

[4] Leiter J.C. (1996). Upper Airway Shape: Is It Important in the Pathogenesis of Obstructive Sleep Apnea?. Am. J. Respir. Crit. Care Med..

[5] Fleury Curado T., Oliven A., Sennes L.U., Polotsky V.Y., Eisele D., Schwartz A.R. (2018). Neurostimulation Treatment of OSA. Chest.

[6] Patil S.P., Schneider H., Marx J.J., Gladmon E., Schwartz A.R., Smith P.L. (2007). Neuromechanical Control of Upper Airway Patency during Sleep. J. Appl. Physiol..

[7] Jordan A.S., White D.P., Owens R.L., Eckert D.J., Rahangdale S., Yim-Yeh S., Malhotra A. (2010). The Effect of Increased Genioglossus Activity and End-Expiratory Lung Volume on Pharyngeal Collapse. J. Appl. Physiol..

[8] Strollo P.J., Gillespie M.B., Soose R.J., Maurer J.T., de Vries N., Cornelius J., Hanson R.D., Padhya T.A., Steward D.L., Woodson B.T. (2015). Upper Airway Stimulation for Obstructive Sleep Apnea: Durability of the Treatment Effect at 18 Months. Sleep.

[9] Serghani M., Heiser C., Schwartz A.R., Amatoury J. (2024). Exploring Hypoglossal Nerve Stimulation Therapy for Obstructive Sleep Apnea: A Comprehensive Review of Clinical and Physiological Upper Airway Outcomes. Sleep Med. Rev..

[10] Alapati R., Wagoner S.F., Nieves A.B., Lawrence A., Rouse D., Larsen C. (2024). Upper Airway Stimulation Device Failure: A 7-Year Single Center Experience. Am. J. Otolaryngol..

[11] Miki H., Hida W., Inoue H., Takishima T. (1988). A New Treatment for Obstructive Sleep Apnea Syndrome by Electrical Stimulation of Submental Region. Tohoku J. Exp. Med..

[12] Decker M.J., Haaga J., Arnold J.L., Atzberger D., Strohl K.P. (1993). Functional Electrical Stimulation and Respiration during Sleep. J. Appl. Physiol..

[13] Strollo P.J., Soose R.J., Maurer J.T., de Vries N., Cornelius J., Froymovich O., Hanson R.D., Padhya T.A., Steward D.L., Gillespie M.B. (2014). Upper-Airway Stimulation for Obstructive Sleep Apnea. N. Engl. J. Med..

[14] Woodson B.T., Strohl K.P., Soose R.J., Gillespie M.B., Maurer J.T., de Vries N., Padhya T.A., Badr M.S., Lin H., Vanderveken O.M. (2018). Upper Airway Stimulation for Obstructive Sleep Apnea: 5-Year Outcomes. Otolaryngol.–Head Neck Surg..

[15] Eastwood P.R., Barnes M., MacKay S., Wheatley J.R., Hillman D.R., Nguyên X.L., Lewis R., Campbell M.C., Pételle B., Walsh J.H. (2019). Bilateral Hypoglossal Nerve Stimulation for Treatment of Adult Obstructive Sleep Apnoea. Eur. Respir. J..

[16] Certal V.F., Zaghi S., Riaz M., Vieira A.S., Pinheiro C.T., Kushida C., Capasso R., Camacho M. (2014). Hypoglossal Nerve Stimulation in the Treatment of Obstructive Sleep Apnea: A Systematic Review and Meta-Analysis. Laryngoscope.

[17] Wray C.M., Thaler E.R. (2016). Hypoglossal Nerve Stimulation for Obstructive Sleep Apnea: A Review of the Literature. World J. Otorhinolaryngol.-Head Neck Surg..

[18] Schwartz A.R., Jacobowitz O., Eisele D.W., Mickelson S.A., Miller M.B., Oliven A., Certal V., Hopp M.L., Winslow D.H., Huntley T.C. (2023). Targeted Hypoglossal Nerve Stimulation for Patients with Obstructive Sleep Apnea. JAMA Otolaryngol.—Head Neck Surg..

[19] Miki H., Hida W., Shindoh C., Kikuchi Y., Chonan T., Taguchi O., Inoue H., Takishima T. (1989). Effects of Electrical Stimulation of the Genioglossus on Upper Airway Resistance in Anesthetized Dogs. Am. Rev. Respir. Dis..

[20] Baptista P.M., Costantino A., Moffa A., Rinaldi V., Casale M. (2020). Hypoglossal Nerve Stimulation in the Treatment of Obstructive Sleep Apnea: Patient Selection and New Perspectives. Nat. Sci. Sleep.

[21] Kezirian E.J., Hohenhorst W., de Vries N. (2011). Drug-Induced Sleep Endoscopy: The VOTE Classification. Eur. Arch. Oto-Rhino-Laryngol..

[22] Vanderveken O.M., Maurer J.T., Hohenhorst W., Hamans E., Lin H.-S., Vroegop A.V., Anders C., de Vries N., Van de Heyning P.H. (2013). Evaluation of Drug-Induced Sleep Endoscopy as a Patient Selection Tool for Implanted Upper Airway Stimulation for Obstructive Sleep Apnea. J. Clin. Sleep Med..

[23] Ravesloot M.J.L., de Vries N. (2011). One Hundred Consecutive Patients Undergoing Drug-Induced Sleep Endoscopy: Results and Evaluation. Laryngoscope.

[24] Kent D.T., Zealear D., Schwartz A.R. (2021). Ansa Cervicalis Stimulation. Chest.

[25] Guilleminault C., Hill M.W., Simmons F.B., Dement W.C. (1978). Obstructive Sleep Apnea: Electromyographic and Fiberoptic Studies. Exp. Neurol..

[26] Kent D.T., Ceremsak J.J., Li Y., Yalamanchi P., Mannion K., Zealear D., Shotwell M.S., Hall M.E., Lindsell C.J., Budnick H.A. (2025). Role of Glossopharyngeal Nerve Stimulation in Stabilizing the Lateral Pharyngeal Wall and Ventilation in OSA: A Pilot Study. Chest.

[27] Alrubasy W.A., Abuawwad M.T., Taha M.J.J., Khurais M., Sayed M.S., Dahik A.M., Keshk N., Abdelhadi S., Serhan H.A. (2024). Hypoglossal Nerve Stimulation for Obstructive Sleep Apnea in Adults: An Updated Systematic Review and Meta-Analysis. Respir. Med..

[28] Wesson T., Rone V., Ramirez M., Manchanda S., Stahl S., Chernyak Y., Parker N. (2024). Outcome Reporting in Prospective Studies Evaluating Neurostimulation for Obstructive Sleep Apnea. Laryngoscope.

[29] Pordzik J., Ludwig K., Seifen C., Ruckes C., Huppertz T., Bahr-Hamm K., Hackenberg B., Matthias C., Gouveris H. (2024). Real-World Data on Polysomnography- and Patient-Reported Outcomes in Hypoglossal Nerve Stimulation and Auto-Titrating Positive Airway Pressure Therapy for Obstructive Sleep Apnea. Respir. Med..

[30] Crossley J.R., Wallerius K., Hoa M., Davidson B., Giurintano J.P. (2021). Association between Conflict of Interest and Published Position on Hypoglossal Nerve Stimulation for Sleep Apnea. Otolaryngology.

[31] Wollny M., Heiser C., Sommer U., Schöbel C., Braun M. (2024). Adverse Events with Hypoglossal Nerve Stimulation in the Treatment of Obstructive Sleep Apnea—A Systematic Review of Clinical Trials and Real-World Data. J. Clin. Med..

